# Disease accumulation across birth cohorts in South Korea

**DOI:** 10.1093/geronb/gbaf136

**Published:** 2025-07-25

**Authors:** Anastasia Lam, Katherine Keenan, Mikko Myrskylä, Hill Kulu

**Affiliations:** Max Planck Institute for Demographic Research, Rostock, Germany; School of Geography and Sustainable Development, University of St Andrews, St Andrews, United Kingdom; School of Geography and Sustainable Development, University of St Andrews, St Andrews, United Kingdom; Max Planck Institute for Demographic Research, Rostock, Germany; Helsinki Institute for Demography and Population Health, University of Helsinki, Helsinki, Finland; School of Geography and Sustainable Development, University of St Andrews, St Andrews, United Kingdom

**Keywords:** health disparities, population aging, longitudinal methods, cohort analysis, chronic disease

## Abstract

**Objectives:**

This article estimates rates and age-trajectories of disease accumulation across South Korean birth cohorts and assesses whether observed cohort differences persist after accounting for early-life exposures and adult characteristics.

**Methods:**

Data were from eight waves of the Korean Longitudinal Study of Aging (2006–2020) and included 8,202 participants aged 50 to 74 years. Birth cohorts were defined by historical periods: Japanese annexation (1932–1944), Korean liberation (1945–1949), Korean War (1950–1953), and Post-war (1954–1961). Poisson mixed-effects models were used to estimate disease accumulation using counts of self-reported chronic diseases. Models were built stepwise from a baseline model with cohort fixed effects. Subsequent models added a cohort/age interaction, early-life exposures (parental death and education), and adult characteristics (own education, residence, smoking, and obesity).

**Results:**

The Post-war (1954–1961) cohort has lower rates of disease accumulation than the older cohorts, which do not differ substantially from each other. Controlling for early-life exposures slightly reduces cohort differences, but controlling for adult characteristics leads to a larger reduction, leaving only the Korean liberation (1945–1949) cohort significantly different from the Post-war (1954–1961) cohort (IRR: 1.17, 95% CI: 1.07–1.29). This suggests adult characteristics explain most of the observed differences between the Post-war (1954–1961) and older cohorts, except the Korean liberation (1945–1949) cohort, which remains uniquely different due to unobserved factors.

**Discussion:**

These findings highlight how historical context and life course experiences are jointly associated with disease accumulation across cohorts. Adult characteristics play an important role, especially for older cohorts, and could be considered as important targets for disease prevention strategies.

Over the 20th century, mortality has decreased substantially due to significant improvements in living standards, economic conditions, and health and social care. However, many studies from the United States and Europe have found that while younger birth cohorts are living longer, they are not necessarily living healthier. Period studies have identified increasing trends in chronic disease and multimorbidity prevalence since the 1990s, which are likely related to changing lifestyle and socioeconomic factors (van Oostrom et al., 2016; Quiñones et al., 2019; Souza et al., 2021). Similarly, cohort studies have found that younger cohorts, particularly those born after the 1940s, show worsening health trends compared to older cohorts (Bishop et al., 2022; Gimeno et al., 2024; Ribe et al., 2024; Zheng et al., 2023). A Canadian study using a hierarchical age-period-cohort (HAPC) approach found evidence for increasing prevalence of chronic disease and multimorbidity over time and among younger birth cohorts (Canizares et al., 2018). However, HAPC studies from Hong Kong and South Korea identified less consistent trends. Compared to older and younger cohorts, birth cohorts from the 1950s in Hong Kong have a lower risk of accumulating disease (Lai et al., 2019), while in South Korea, birth cohorts from the 1930s and 1940s have a higher risk of diabetes and hypertension relative to more recently born cohorts (Namkung & Kang, 2024).

While a period perspective is more common and is useful for understanding changes over a particular time period, e.g., between 2000 and 2020, or in response to specific events, e.g., the COVID-19 pandemic, a cohort comparison perspective is better suited for identifying long-term impacts of historical events and shifts in social norms (Dattani & Roser, 2023; Jones et al., 2023). Thus, this study will compare birth cohorts defined by the sociohistorical context of South Korea and interpret cohort differences within a life course framework.

South Korea provides a unique opportunity to study cohort patterns in health because major political and cultural changes, war, and economic development have occurred in a relatively narrow period of time. This means that each cohort experienced the same substantial social and economic changes at different stages of their life course, which may explain variations in their later-life health. Additionally, South Korea’s rapid population aging and low fertility make it a relevant setting to understand how health patterns are changing with successive cohorts in light of individuals’ characteristics.

To gain a better understanding of how historical context and life course exposures are associated with later-life health, this study addresses the following research questions:

- How does chronic disease accumulation differ across birth cohorts defined by major cultural, political, and economic changes in South Korean history?
- Do observed cohort differences in disease accumulation persist after accounting for early-life exposures and adult characteristics?

## Background

### A life course approach for studying cohort differences

The life course approach provides a framework to understand cross-cohort disease accumulation patterns by emphasizing the importance of sociohistorical environment and temporal processes in shaping health trajectories. In particular, Elder’s life course principle of time and place stresses the influence of historical processes in specific geographic places for shaping unique cohort experiences (Elder et al., 2003). These factors could systematically alter life trajectories and provide important context for understanding cohort differences in health.

South Korea’s recent history is characterized by political, social, and economic changes, which likely play important roles in shaping the life courses of different birth cohorts. Individuals born during Japanese annexation (1910–1945) faced substantial cultural oppression, being forced to adopt Japanese customs, language, and policies in lieu of their own (Cumings, 2021). Those born after Korea gained its independence in 1945 witnessed its division into North and South Korea, at a time when North Korea was more industrially developed and South Korea more agricultural (Seth, 2017). Following this period, the Korean War, which lasted from 1950 to 1953, had detrimental effects on South Korea’s population, infrastructure, and economy, making it one of the poorest countries in the world (Kim, 2019). Individuals living during this period experienced food shortages and the destruction of most existing infrastructure, and half the population lived in extreme poverty. However, from 1961, the South Korean economy began to rapidly develop, transitioning to a middle-income country by the mid-1980s and a high-income country in 1996 (Kim, 2019). Based on these periods, we define the following birth cohorts: Japanese annexation (1932–1944), Korean liberation (1945–1949), Korean War (1950–1953) and Post-war (1954–1961).

Life course epidemiology models suggest that social and biological exposures during early life and adulthood combine in complex ways to have long-term effects on later-life health (Kuh et al., 2003). The critical period model proposes that adverse events (e.g., parental death) occurring during key developmental stages will lead to adverse health later in life (Kuh et al., 2003; Luecken & Roubinov, 2012). In our study, members of the Korean liberation (1945–1949) and Korean War (1950–1953) cohorts were either infants or still in-utero when they were exposed to wartime stress, potentially putting them at higher risk of developing cardiometabolic conditions, musculoskeletal problems, and functional limitations compared to individuals from the Japanese annexation (1932–1944) cohort who were exposed to such stresses during later stages of childhood or adolescence (Haas & Ramirez, 2022; Lee, 2014, 2017; Ramirez & Haas, 2022).

In addition to adverse early-life events, lower parental education—an important indicator of childhood socioeconomic status—is associated with poorer self-rated health and lower life expectancy in older age (Huebener, 2019; Lee et al., 2020). Due to educational expansion since the Japanese annexation, we expect that the meaning of parental education differs across cohorts. For example, members of older cohorts are more likely to have parents with little or no formal education, since formal schooling only started after 1910 (Chang, 1975). In contrast, the Post-war (1954–1961) cohort likely has parents (mainly fathers) with higher levels of education. This educational expansion not only benefited parental cohorts but also contributed to the increasing levels of educational attainment across the cohorts in this study. Therefore, studying how disease accumulates differently across these cohorts offers valuable insights into how the timing of historical context and life course experiences may contribute to later-life health.

### Cross-cohort health patterns in South Korea

Over the 20th century, South Korea and other middle-income countries experienced rapid economic, demographic, and health transitions, thus exposing cohorts to several stages of health transition in a relatively short period (Vallin & Meslé, 2004). South Korea’s rapid economic development was accompanied by increases in urbanization and educational attainment. From the end of the Korean War to the 2000s, the percentage of people living in rural areas declined from about 70% to 20% (Ma et al., 2018). Young adults (ages 10–29) were the most mobile, migrating to urban areas for better job opportunities, education, and marriage (Kim, 1982). During this same period, educational attainment soared, rising from 65% of children receiving primary education in 1945 to over 90% receiving at least a secondary school education in 2010 (Kim & Lee, 2010). These developments allowed the Post-war (1954–1961) cohort access to better resources at younger ages, such as improved health care access and more job opportunities, but the environmental and lifestyle changes associated with urbanization may put them more at risk of poorer health and chronic diseases compared to older cohorts.

As mortality and fertility rates declined due to improvements in living conditions and medical advancements, there was also a clear progression through the stages of Vallin & Meslé’s (2004) health transition. In 1990, the top five causes of death were stroke, ischemic heart disease, cirrhosis, stomach cancer, and road injuries; the latter three were replaced by lung cancer, Alzheimer’s disease, and lower respiratory infections in 2019, respectively (Park et al., 2023). Obesity is a well-known risk factor for cardiometabolic diseases, such as stroke and ischemic heart disease, and its prevalence is increasing over time across all age groups in South Korea (Yang et al., 2022). The prevalence of obesity in 2009 for people in their 50s, 60s, and 70s was 36%, 38%, and 34%, respectively. By 2019, this increased only two percentage points for those in their 50s and 60s and up to seven percentage points for those in their 70s (Yang et al., 2022). Based on these estimates, we would expect younger cohorts to have a higher baseline prevalence of obesity compared to older cohorts, but differences should be minor. The rise of lung cancer and lower respiratory infections as leading causes of death is likely related to high rates of smoking from the 1970s to 1990s (Choi et al., 2007) and increasing air pollution in urban areas (Kim et al., 2021). Therefore, we would expect that the younger cohorts, particularly the Post-war (1954–1961) cohort, will have a higher prevalence of smoking and lung problems compared to the others (Kim et al., 2022).

### Summary and hypotheses

Over the last century, Korea has transitioned through states of colonization, liberation, war, and industrialization. Now, it is facing the challenge of population aging. By comparing cohorts born during Japanese annexation (1932–1944), Korean liberation (1945–1949), Korean War (1950–1953), and Post-war (1954–1961), this study investigates how disease accumulation differs across birth cohorts in South Korea and whether early-life and adult exposures are associated with cohort differences.

We hypothesize that the Korean liberation (1945–1949) and Korean War (1950–1953) cohorts will have the highest rate of disease accumulation because of their early life and in-utero exposure to the Korean War. In contrast, the Post-war (1954–1961) cohort will likely accumulate disease more slowly than the other cohorts because they had access to better living and economic conditions earlier in their life, and improved disease screening and prevention measures to mitigate disease development. However, these advantages may be offset by a generational health drift identified in other high-income countries (Gimeno et al., 2024), which suggests that younger cohorts will have more disease than older cohorts because of urbanization and changing lifestyles.

## Method

### Data

This study uses data from the Korean Longitudinal Study of Aging (KLoSA) (Korea Employment Information Service, 2015). The KLoSA collects information on various socioeconomic, demographic, and health factors for participants aged 45 years and older across South Korea, excluding Jeju Island (Korea Employment Information Service, 2023). At Wave 1 (2006), 10,254 participants were interviewed and followed up biannually to Wave 8 (2020). The attrition rate after the first wave was 13.4% and gradually increased across waves to 22.9% at Wave 8 (Korea Employment Information Service, 2023). We excluded observations (i.e., waves) where the participant was not interviewed (22,184 observations). We then excluded individuals who were missing information across all waves on their own education (*n* = 8; 32 observations), all included diseases (*n* = 3; 3 observations), parental education (*n* = 82; 339 observations), and baseline height and/or weight (*n* = 134; 465 observations). We additionally excluded 1,826 individuals (18,320 observations) while they were under age 50 and over age 74 to have an age range that was more evenly distributed across the different birth cohorts. Individuals needed to have at least one observation and could enter the study after Wave 1 if they became age-eligible. Supplementary Material, Section 1 provides further details of excluded individuals and observations. Our final analytic sample consisted of 8,202 individuals (40,689 observations) who were present in at least one wave. The total number of individuals and observations for each birth cohort can be seen in Table 1.

**Table 1.** Characteristics of study sample at participant’s entry wave, by cohort. Data are from the Korean Longitudinal Study of Aging (2006–2020).

Variable	Japanese annexation (1932–1944) (*n* = 3,382)	Korean liberation (1945–1949) (*n* = 1,376)	Korean War (1950–1953) (*n* = 1,112)	Post-war (1954–1961) (*n* = 2,332)	***p*** ^a^
Total observations over follow-up	11,465	8,744	7,208	13,272	<.001
Mean observations per person	3.39	6.35	6.48	5.69	
Disease count					
0	1,267 (37.5%)	729 (53.0%)	698 (62.8%)	1,677 (71.9%)	<.001
1	1,209 (35.7%)	408 (29.7%)	285 (25.6%)	507 (21.7%)	
2	609 (18.0%)	170 (12.4%)	100 (9.0%)	125 (5.4%)	
3	228 (6.7%)	52 (3.8%)	27 (2.4%)	20 (0.9%)	
4	62 (1.8%)	13 (0.9%)	1 (0.1%)	3 (0.1%)	
5	5 (0.1%)	3 (0.2%)	0 (0%)	0 (0%)	
6	2 (0.1%)	1 (0.1%)	0 (0%)	0 (0%)	
7	0 (0%)	0 (0%)	1 (0.1%)	0 (0%)	
Sex					
Male	1,522 (45.0%)	665 (48.3%)	505 (45.4%)	1,008 (43.2%)	.0272
Female	1,860 (55.0%)	711 (51.7%)	607 (54.6%)	1,324 (56.8%)	
Age (years), Mean (*SD*)	67.6 (3.54)	58.9 (1.40)	54.5 (1.12)	50.9 (1.03)	<.001
Parental death during childhood					
No	2,932 (86.7%)	1,197 (87.0%)	1,008 (90.6%)	2,118 (90.8%)	<.001
Yes	450 (13.3%)	179 (13.0%)	104 (9.4%)	214 (9.2%)	
Parental education					
No formal education	2,379 (70.3%)	817 (59.4%)	598 (53.8%)	901 (38.6%)	<.001
Elementary school	740 (21.9%)	395 (28.7%)	369 (33.2%)	885 (38.0%)	
Middle school or more	263 (7.8%)	164 (11.9%)	145 (13.0%)	546 (23.4%)	
Own education					
Middle school or less	2,620 (77.5%)	888 (64.5%)	612 (55.0%)	774 (33.2%)	<.001
High school	536 (15.8%)	362 (26.3%)	377 (33.9%)	1,139 (48.8%)	
College/university	226 (6.7%)	126 (9.2%)	123 (11.1%)	419 (18.0%)	
Urban/rural					
Urban	2,458 (72.7%)	1,056 (76.7%)	914 (82.2%)	1,953 (83.7%)	<.001
Rural	924 (27.3%)	320 (23.3%)	198 (17.8%)	379 (16.3%)	
Smoking status					
Ever smoker	960 (28.4%)	443 (32.2%)	349 (31.4%)	676 (29.0%)	.0299
Never smoker	2,422 (71.6%)	933 (67.8%)	763 (68.6%)	1,656 (71.0%)	
Obesity status					
Obese	811 (24.0%)	363 (26.4%)	317 (28.5%)	534 (22.9%)	.0013
Not obese	2,571 (76.0%)	1,013 (73.6%)	795 (71.5%)	1,798 (77.1%)	

a *p*-value computed using Pearson Chi-square test, except for “Disease count,” which used Fisher exact test due to small sample sizes. H0 = there are no differences between cohorts.

**Figure 1.**
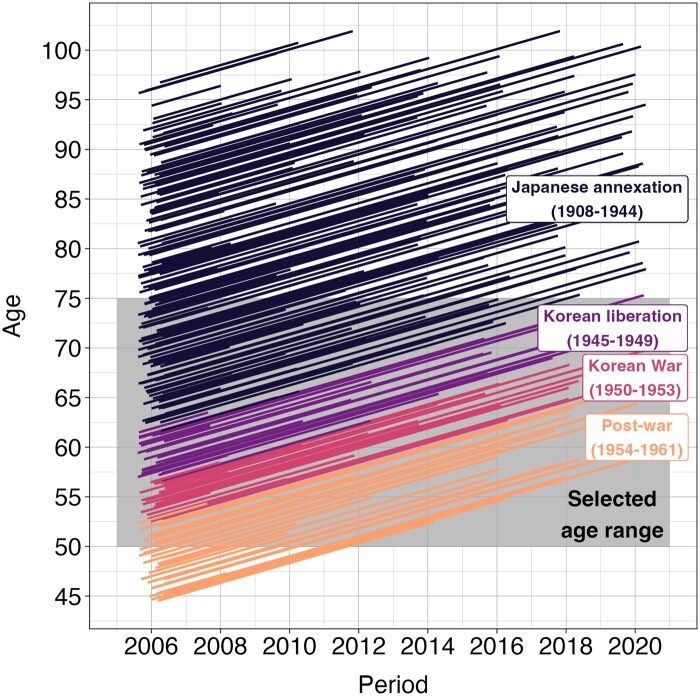
Lexis diagram depicting entry and follow-up times for a sample (*n* = 250) of participants from the original study population, prior to any exclusions. The grey area represents the age range selected for our final analytic sample. Data are from the Korean Longitudinal Study of Aging (2006–2020).

Figure 1 uses a lexis diagram to depict the entry and ­follow-up time for a sample of participants. Members of each cohort can enter and exit the study at any age between 50 and 74 years, with each cohort entering the study at a different age. In Figure 1, the Japanese annexation cohort includes individuals born between 1908 and 1944 because this is derived from the initial sample before any exclusions, whereas the analytic sample only includes those individuals born between 1932 and 1944.

### Outcome

The outcome is disease count, defined as counts of the following self-reported diseases: arthritis, cancer, chronic lung disease, diabetes, heart disease, hypertension, liver disease, psychiatric disorders, and stroke. These diseases were chosen because first, they were asked about in all study waves, and second, because they cover the major causes of death and disability in South Korea (Vos et al., 2020). Additionally, they cover many of the core conditions, which should be included in measures of multimorbidity (Ho et al., 2021). Participants were asked whether they had ever been diagnosed with one of these diseases. If the participant answered “yes,” they were not asked again in subsequent waves because that value was carried forward, indicating the chronic nature of these diseases.

### Predictors

We use four distinct cohorts in our analysis derived from self-reported year of birth: Japanese annexation (1932–1944), Korean liberation (1945–1949), Korean War (1950–1953), and Post-war (1954–1961). The following measures were also self-reported. Age in years is used as a continuous variable. Sex is categorized as male or female. Parental death during childhood is defined as whether or not at least one parent died while the participant was under 15 years old. Parental education is defined as the highest level of education obtained by either mother or father and categorized as “no formal education,” “elementary school,” or “middle school or more.” Own education is the participant’s highest level of reported education, categorized as “middle school or less,” “high school,” and “university.” Geography is time-varying and dichotomized as urban or rural residence at the time of the survey. Although smoking status and obesity are known to change across the life course, they are also both risk factors and consequences of various chronic diseases. Therefore, we used baseline values in the analysis to minimize the potential bias due to reverse causation with our outcome. Smoking is defined as whether the participant was an ever/current or never smoker. Obesity is defined using the World Health Organization’s recommended body mass index (BMI) cutoff of ≥25kg/m^2^ for Asia-Pacific populations (World Health Organization. Regional Office for the Western Pacific, 2000). We used the BMI values that KLoSA calculated based on respondents’ reported height and weight for waves one to seven. For three individuals whose baseline was Wave 8, we calculated BMI based on their reported height and weight.

### Statistical analysis

We computed descriptive statistics for the total study sample by birth cohort. We also computed age-specific prevalence of disease, calculated as the observed disease count per age and birth cohort divided by the total number of observations per age and birth cohort, and calculated the proportion of each disease distributed across birth cohorts. Poisson mixed-effects models, with repeated measures (Level 1) nested within individuals (Level 2), were used to estimate the incidence of disease accumulation and predict disease count across age and cohort (Bolker et al., 2009). Models were built in a stepwise manner, starting with Model 1, which contains the outcome (disease count) and fixed effects for cohort, sex, and age. Model 2 adds an interaction term between cohort and age. Model 3 extends Model 2 by adding early-life exposures (parental death during childhood and parental education). Model 4 extends Model 2 by adding adult characteristics (own education, geography, smoking, and obesity). Model 5 is the fully-adjusted model which includes all early-life and adult predictors.

Statistical analyses were conducted in R Studio (R version 4.2.0) (RStudio Team, 2023).

### Sensitivity analysis

First, due to well-known sex differences in health and health behaviors, we stratified models by sex. Second, due to the uneven distribution of diseases across cohorts, we wanted to examine whether different types of diseases might help explain the observed cohort differences. Therefore, we grouped diseases into low-mortality (arthritis and hypertension) or high-­mortality (cancer, chronic lung disease, diabetes, heart disease, liver disease, and stroke) based on whether they were among the leading causes of death in South Korea in 2021 (Institute for Health Metrics and Evaluation (IHME), 2024). Psychiatric disorders can include a wide range of diseases, so we included them in both low-mortality and high-mortality groups. Further, most studies include hypertension in multimorbidity (Ho et al., 2021), but there remains debate about whether it is a risk factor or a chronic disease. Arthritis is a chronic disease, but it is less associated with mortality than other diseases such as cancer or stroke. Thus, we wanted to additionally evaluate whether excluding hypertension and/or arthritis would alter our findings.

Third, to examine the role of excess extrapolation due to uneven participant distribution at the youngest and oldest ages, we ran the analysis for different age ranges: 50–70 years, 55–70 years, and 55–75 years.

Fourth, dementia was only assessed in the last two study waves, and thus was not included in our outcome. We included the Mini-Mental State Examination (MMSE) score as a proxy to test whether excluding neurodegenerative issues might be driving the lower multimorbidity prevalence of the Japanese annexation (1932–1944) cohort.

Lastly, as a robustness check, we performed sensitivity analysis excluding obesity from the models. Different cohorts enter the study at different ages, and BMI is measured at entry. However, BMI tends to change over age, which makes it difficult to compare across cohorts.

## Results

### Descriptive statistics

Table 1 provides descriptive characteristics by cohort for the first wave when an individual enters the analysis (when they are at least 50 years old). The Post-war (1954–1961) cohort had the most observations over follow-up (13,272), and the Korean War (1950–1953) cohort had the highest average number of observations per person (6.48). Disease count was lower in the younger cohorts, with 72% of the Post-war (1954–1961) cohort having no disease compared to 38% of the Japanese annexation (1932–1944) cohort. Females comprised over half the sample in each cohort. The average age was 67.6, 58.9, 54.5, and 50.9 years in the Japanese annexation (1932–1944), Korean liberation (1945–1949), Korean War (1950–1953), and Post-war (1954–1961) cohorts, respectively. A greater proportion of individuals from the Japanese annexation (1932–1944) and Korean liberation (1945–1949) cohorts lost a parent during childhood compared to the more recent cohorts (13% vs 9%). The older cohorts had a greater proportion of low parental and their own education. There was also an increasing trend in urban residence across cohorts. For smoking and obesity status, the two middle cohorts had the highest percentages of ever smoking and being obese.

### Age-specific prevalence of disease

**Figure 2.**
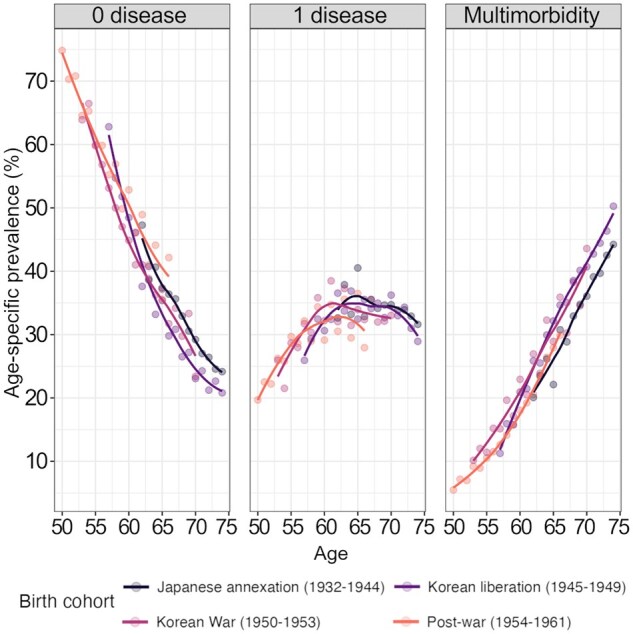
Age-specific prevalence of zero disease, one disease, and multimorbidity for each birth cohort. Lines are smoothed across points using the LOESS method. Data are from the Korean Longitudinal Study of Aging (2006–2020).

For all cohorts, the fraction of individuals with no disease decreased with age, the proportion of one disease increased until the early 60s then flattened, and the proportion of multimorbidity increased with age (Figure 2). It was difficult to discern differences between cohorts due to intersecting lines and different age ranges. However, we observed that the Japanese annexation (1932–1944) and Korean liberation (1945–1949) cohorts had slightly higher prevalence of one disease than the younger cohorts from around age 65. The Korean liberation (1945–1949) and Korean War (1950–1953) cohorts had a relatively higher prevalence of multimorbidity than the Japanese annexation (1932–1944) and Post-war (1954–1961) cohorts from age 60 years.

### Distribution of diseases across birth cohorts

The distribution of diseases by birth cohort and sex for ages 62 to 66 years, reflecting the ages in which all cohort groups are represented, is presented in Supplementary Material, Section 2. Hypertension was the most common condition, followed by arthritis (females) and diabetes (males). The Japanese annexation (1932–1944) cohort had the highest prevalence of diabetes, heart disease, and lung disease, the Korean liberation (1945–1949) cohort had the highest prevalence of hypertension, arthritis, and stroke, the Korean War (1950–1953) cohort had the highest prevalence of liver disease and psychiatric disorders, and the Post-war (1954–1961) cohort had the highest prevalence of cancer.

### Observed cohort differences in disease accumulation rate

Table 2 presents results from the Poisson mixed-effects models, which estimated the associations between disease count, birth cohort, early-life exposures, and adult characteristics. Both age and being female were associated with higher rates of disease accumulation across all models. In Model 1, compared to the Post-war (1954–1961) reference cohort, the Japanese annexation (1932–1944), Korean liberation (1945–1949), and Korean War (1950–1953) cohorts all had higher rates of disease accumulation (Incidence rate ratio (IRR) (95% CI): 1.23 (1.14–1.34), 1.33 (1.21–1.46), and 1.23 (1.12–1.36), respectively). After the age/cohort interaction is added, then for subsequent models, the cohort IRRs reflect cohort differences at the centered age (62.9 years), relative to the reference cohort. In Model 2, although the cohort IRRs were attenuated compared to Model 1, they followed the same pattern, and the interaction terms were significant for all cohorts. This provides some evidence for the first research question that examines how disease accumulation differs across birth cohorts in South Korea. Although the confidence intervals for the older cohorts overlapped, indicating no statistically significant difference between them, they all significantly differed from the Post-war (1954–1961) reference cohort.

**Table 2.** Incidence rate ratios with 95% confidence intervals for disease accumulation across four birth cohorts, adjusted for sex, age, early-life exposures, and adult characteristics. Data are from the Korean Longitudinal Study of Aging (2006–2020).

Variable	Model 1	Model 2	Model 3	Model 4	Model 5
Intercept	0.41 (0.38–0.44)***	0.43 (0.40–0.46)***	0.44 (0.41–0.48)***	0.85 (0.76–0.95)***	0.84 (0.75–0.93)***
Female	1.35 (1.27–1.44)***	1.35 (1.27–1.44)***	1.35 (1.28–1.44)***	1.33 (1.23–1.44)***	1.33 (1.23–1.45)***
Age^a^	1.06 (1.06–1.06)***	1.07 (1.06–1.08)***	1.07 (1.06–1.08)***	1.07 (1.06–1.08)***	1.07 (1.06–1.08)***
Birth cohort					
Japanese annexation (1932–1944)	1.23 (1.14–1.34)***	1.18 (1.08–1.29)***	1.15 (1.04–1.26)**	1.07 (0.97–1.18)	1.07 (0.97–1.17)
Korean liberation (1945–1949)	1.33 (1.21–1.46)***	1.28 (1.17–1.41)***	1.25 (1.14–1.38)***	1.18 (1.07–1.30)**	1.17 (1.07–1.29)**
Korean War (1950–1953)	1.23 (1.12–1.36)***	1.17 (1.06–1.30)**	1.15 (1.04–1.28)**	1.08 (0.98–1.20)	1.08 (0.97–1.20)
Age:Birth cohort interaction					
Age:Japanese annexation (1932–1944)		0.99 (0.98–1.00)*	0.99 (0.98–1.00)*	0.99 (0.98–1.00)*	0.99 (0.98–1.00)*
Age:Korean liberation (1945–1949)		0.99 (0.98–0.99)***	0.99 (0.98–0.99)***	0.99 (0.98–0.99)**	0.99 (0.98–0.99)**
Age:Korean War (1950–1953)		0.99 (0.98–1.00)*	0.99 (0.98–1.00)*	0.99 (0.98–1.00)*	0.99 (0.98–1.00)*
Parental death during childhood			1.13 (1.03–1.24)**		1.09 (1.00–1.19)
Parental education					
Elementary school			0.96 (0.90–1.03)		1.01 (0.94–1.09)
Middle school or more			0.87 (0.79–0.96)**		0.99 (0.90–1.09)
Own education					
High school				0.81 (0.76–0.87)***	0.81 (0.76–0.88)***
College/university				0.70 (0.63–0.78)***	0.71 (0.63–0.79)***
Rural residence				0.91 (0.84–0.96)**	0.91 (0.86–0.97)**
Ever smoker				1.15 (1.06–1.25)**	1.15 (1.06–1.25)**
Obese				1.68 (1.58–1.79)***	1.68 (1.58–1.79)***
Random intercept variance	1.31	1.31	1.31	1.22	1.22
Intraclass correlation coefficient (ICC)	0.57	0.57	0.57	0.56	0.56

*Note.* Results were computed using Poisson mixed-effects models with repeated measures at level 1 and individuals at level 2. Due to rounding, some confidence intervals appear to include 1.00 but remain statistically significant.

a Age is grand-mean centered at 62.9 years.

* *p *< .05.

* *p *< .01.

* *p *< .001.

Once early-life exposures were controlled for in Model 3, the birth cohort IRRs decreased slightly but remained statistically significantly different from the Post-war (1954–1961) cohort at the centered age (Table 2). This could indicate that the older cohorts’ higher rate of disease accumulation is partially explained by early-life exposures, such as parental death during childhood or low parental education. In other words, if the Post-war (1954–1961) cohort experienced the same early-life disadvantages as the older cohorts, their rate of disease accumulation would be more similar, thus narrowing the cohort differences.

When adult characteristics were controlled for in Model 4, we observed a larger attenuation of the birth cohort IRRs, and only the Korean liberation (1945–1949) cohort remained statistically significantly different from the reference cohort at the centered age (Table 2). This pattern remained in the fully-­adjusted model (Model 5), providing evidence that some of the observed cohort differences persist after accounting for early-life exposures and adult characteristics.

### Difference in predicted disease count by age and birth cohort

**Figure 3.**
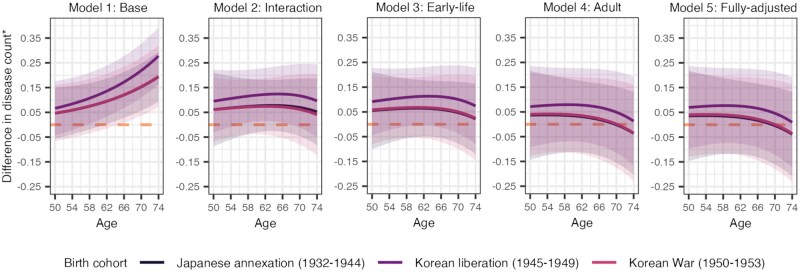
Difference in predicted disease counts for each cohort relative to the Post-war (1954–1961) cohort (dashed orange line), based on Models 1–5. Data are from the Korean Longitudinal Study of Aging (2006–2020). *Difference in disease count calculated by subtracting the predicted disease count of the Post-war (1954–1961) cohort from the predicted disease count of each of the other cohorts.

Figure 3 shows the difference in predicted disease counts for each cohort relative to the Post-war (1954–1961) cohort based on Models 1–5. Confidence intervals for all cohorts overlapped and crossed the reference line, indicating no significant differences in predicted disease count between cohorts and compared to the Post-war (1954–1961) cohort. However, we can observe that in the panel for Model 1, the differences in predicted disease count increased with age for all cohorts, but once the age/cohort interaction was added in Model 2, the age effect seemed to reverse and flatten. Little change occurred once early-life exposures were added in Model 3, but when adult characteristics were added in Model 4, the difference in predicted disease count decreased slightly for all cohorts, thereby narrowing the gap with the Post-war (1954–1961) cohort. Additionally, after age 70, the difference in predicted disease count between the Post-war (1954–1961) cohort and the Japanese annexation (1932–1944) and Korean War (1950–1953) cohorts was negative, meaning that the older cohorts appear to have a lower predicted disease count than the youngest cohort.

### Supplementary analyses

Sex-stratified models showed no statistically significant cohort differences among males. Females had the same pattern as the main analysis, where the Korean liberation (1945–1949) cohort was statistically significantly different from the Post-war (1954–1961) cohort (Supplementary Material, Section 3). For low-mortality diseases, all cohorts differed significantly from the Post-war (1954–1961) cohort, except the Korean War (1950–1953) cohort, when psychiatric disorders were added (Supplementary Table 3 in Supplementary Material, Section 4). There were no significant cohort differences between the Post-war (1954–1961) and older cohorts for high-mortality diseases. Other sensitivity analyses found patterns similar to the main analysis (Supplementary Material, Section 4).

## Discussion and conclusion

In this article, we investigated whether there are cohort differences in disease accumulation across four contextually distinct birth cohorts in South Korea, and if those observed differences persist after accounting for early-life exposures and adult characteristics. In the unadjusted model, all older cohorts had statistically higher rates of disease accumulation compared to the Post-war (1954–1961) cohort at the sample’s mean age (62.9 years). Controlling for early-life exposures and adult characteristics attenuated these differences, but the Korean liberation (1954–1961) cohort maintained a statistically significantly higher rate of disease accumulation than the Post-war (1954–1961) cohort, highlighting the contribution of unobserved factors. Additionally, we did not identify any significant differences in predicted disease count across cohorts, potentially due to our study being underpowered.

The Korean liberation (1945–1949) cohort’s higher rate of disease accumulation compared to the Post-war (1954–1961) cohort may reflect unobserved life course factors from early life, such as their exposure to the Korean War and its aftermath during a critical development period (ages 1–8). Previous studies have found that early-life exposure to war has adverse effects for later-life health, specifically in terms of the development of chronic diseases, functional and physical limitations, and obesity (Akbulut-Yuksel, 2017; Haas & Ramirez, 2022, 2024). While some studies find the in-utero or infant period to be the most important for later-life health (Lee, 2014, 2017; Ramirez & Haas, 2021), others suggest that war exposure during in-utero and early childhood (e.g., before age 8) periods both have long-term consequences, particularly for cardiometabolic health (Haas & Ramirez, 2022; Han & Hong, 2019).

Early childhood adversity has also been associated with increased risk of psychological conditions (Kessler et al., 2001), decreased educational attainment, and increased risky behavior in later life (Ramirez & Haas, 2021). Because the Korean liberation (1945–1949) and Korean War (1950–1953) cohorts were at different developmental stages during the war, their experiences and later-life health outcomes likely differ. We expected that the Korean War (1950–1953) cohort, who were in-utero or infants during wartime, would be more influenced by war-related risk factors, given the well-established importance of fetal and infant development periods for later-life health (Barker, 1990; Kuh et al., 2003). While prior research has found evidence for this, it is also possible that the Korean liberation (1945–1949) cohort is influenced by war-related risk factors in its own distinct way. For example, unlike those in the Korean War (1950–1953) cohort who probably do not explicitly remember the conflict, those in the Korean liberation (1945–1949) cohort were old enough to experience the stress, undernutrition, and destruction that accompanies war, but too young to properly process their trauma (Ramirez & Haas, 2021). This exposure could have triggered lasting biological or physiological adaptations, which shaped their later-life health (Ben-Shlomo & Kuh, 2002; Haas, 2008). However, it is important to acknowledge that this study does not directly measure war exposure and only assumes differential exposure due to birth cohort. Other unmeasured factors from early life or later in adulthood, such as proximity to conflict zones, postwar socioeconomic conditions, or differences in access to health and social resources over the life course, may also contribute to the patterns we observe. Further research is needed to identify which of these specific mechanisms may be driving the cohort differences.

Members of the Post-war (1954–1961) cohort are distinct from members of the other cohorts because they did not experience the Korean War firsthand during their life course. Although they did experience the postwar aftermath in their early childhood, most of their lives were spent during a period of rapid economic and social development. This allowed them the opportunity to access better resources, such as more education, better nutrition, and improved healthcare, at earlier stages of their life. However, increased urbanization and changing lifestyles may influence both disease accumulation and combinations of diseases (disease profiles). As we do not conduct disease-specific analysis, cohort differences could be explained by shifting disease profiles rather than accumulation alone.

A recent study from South Korea took a HAPC approach to examine trends in the prevalence of diabetes and hypertension among adults aged 65 years and older from 2004 to 2020 (Namkung & Kang, 2024). They identified some period effects, whereby the likelihood of being diagnosed with diabetes and hypertension increased over time. The authors attribute these increasing trends to improvements in health care treatment and screening as well as shifting lifestyle behaviors, which could also explain our findings. However, in terms of cohort effects, Namkung & Kang (2024) identified an inverted U-shape pattern, where their middle birth cohorts (those born between 1935–1939 and 1940–1944) had the highest prevalence of diabetes and hypertension compared to older and younger cohorts. This may differ from our findings for several reasons. First, Namkung & Kang (2024) define cohorts in 5-year intervals from “1924 or earlier” to “1950-1955” to address the identification problem in HAPC models, which differs from our deliberate categorization of cohorts based on distinct historical periods. Therefore, our Japanese annexation (1932–1944) cohort overlaps with three of their cohort groups, which could partly explain the different patterns. Second, the authors focus on the prevalence of diabetes and hypertension as individual diseases, whereas our study moves beyond cardiovascular health to consider disease accumulation of up to eight different chronic diseases, including diabetes and hypertension. While this approach limits our ability to account for shifting disease profiles across cohorts, it captures a more holistic picture of health and aging as processes of accumulation. Figure 2 shows that the Japanese annexation (1932–1944) cohort seems to have slightly higher age-specific prevalence of one disease from age 65 compared to the younger cohorts, which is consistent with Namkung & Kang’s (2024) findings. When multimorbidity is considered, however, the Japanese annexation (1932–1944) cohort appears to have a lower prevalence from age 65 years compared to the younger cohorts, highlighting that different patterns may exist when looking at single versus multiple diseases. Third, Namkung & Kang (2024) use pooled cross-sectional data to estimate population-level temporal trends in chronic diseases across periods and cohorts. In contrast, our study uses longitudinal data to understand ­individual-level changes in disease accumulation and how that differs across cohorts. Although the findings differ between these studies, both provide valuable insights into the sparse field of cohort health differences in South Korea and demonstrate that further research is necessary to understand the macro- and micro-level complexities of chronic disease patterns.

This study has several limitations. First, there is survival bias, in that participants had to have survived through myriad adverse events to participate and remain in the survey and therefore might be characteristically different from people who did not survive to this point. Second, all information was self-reported, which makes the data susceptible to recall bias, particularly for early-life exposures. Third, we did not have information on many relevant early-life variables, such as place of birth, geographic residence during the war, or childhood socioeconomic and health conditions, which could potentially explain some of the observed cohort differences. Fourth, due to a limited sample size of some individual diseases, we could not conduct disease-specific analysis. Thus, it was not possible to assess if changes in the same individual diseases across cohorts may contribute to the cohort differences we observed. Last, due to the sparse/unevenly distributed data at the youngest and oldest ages across cohorts, we could not account for those ages without excess extrapolation. However, the central age range chosen for the analysis is representative of when chronic diseases are most likely to develop (Zhu et al., 2018). Even with the limited age range we chose, the youngest and oldest ages are not fully represented by all cohorts. This adds additional uncertainty to our estimates and necessitates that the difference in predicted disease counts must be interpreted with caution.

Viewing disease accumulation using a life course framework, by jointly considering historical context and life course characteristics, allows for the broader consideration of factors related to successful aging. We identified cohort differences in disease accumulation, which persist after accounting for early-life exposures and adult characteristics, indicating other unobserved components warrant further research. Specifically, investigating potential reasons the Korean liberation (1945–1949) cohort has higher rates of disease accumulation is needed. This could include conducting disease-specific analysis across cohorts or incorporating childhood factors with more direct links to ­later-life health, broader indicators of adult socioeconomic status, and family structure. Additionally, as South Korea’s population ages and disease accumulation rates increase, it is essential to implement preventive measures, especially targeting adult characteristics, and develop a robust health and social care infrastructure that accounts for cohort-specific differences.

## Supplementary Material

gbaf136_Supplementary_Data
