# Supplementary material for: Disease accumulation across birth cohorts in South Korea
Source: J Gerontol B Psychol Sci Soc Sci. 2025 Jul 25;80(11):gbaf136. doi: 10.1093/geronb/gbaf136 (PMC12515078; doi:10.1093/geronb/gbaf136)
Supplement: gbaf136_Supplementary_Data [file gbaf136_supplementary_data.zip › JGSS suppl Lam et al.docx]

***The Journals of Gerontology, Series B: Psychological Sciences and Social Sciences* Supplementary Material: Lam, Keenan, Myrskylä, & Kulu. Disease accumulation across birth cohorts in South Korea.**

**Section 1: Supplementary Figure 1.** Sample selection flowcharts, by number of observations and individuals

**
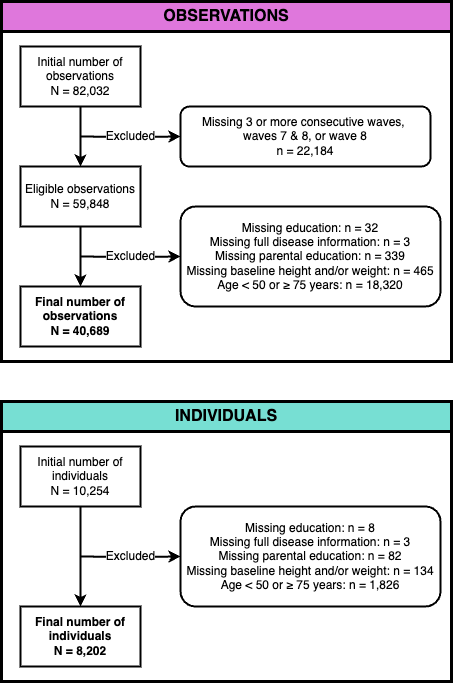
**

**Section 2: Supplementary Figure 2.** Disease distribution across birth cohorts, combined and by sex

| **(A)** | **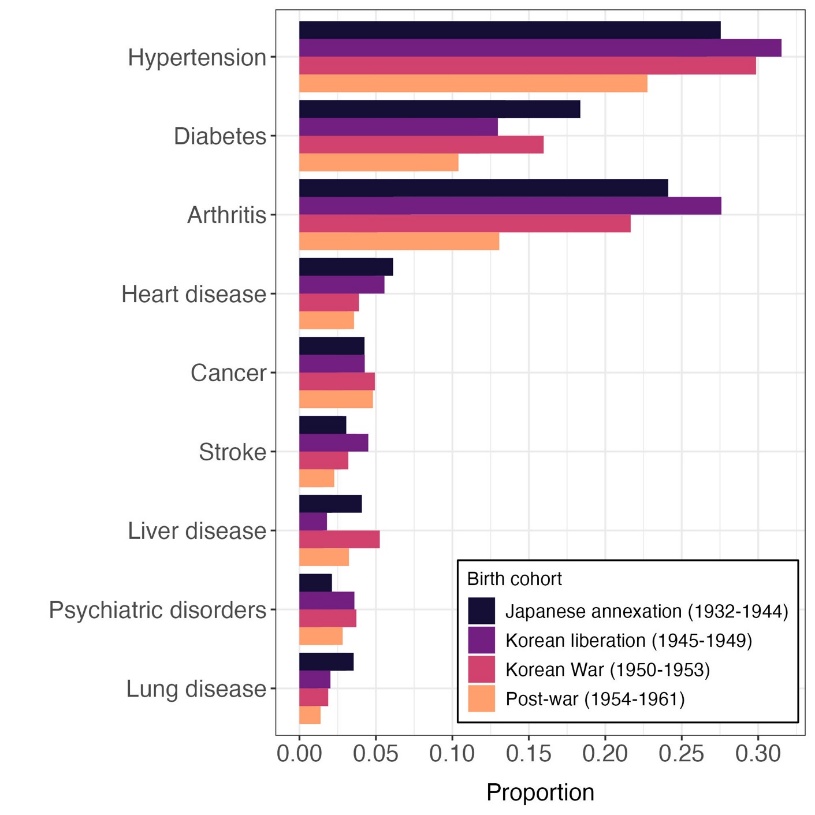** |
| --- | --- |
| **(B)** | 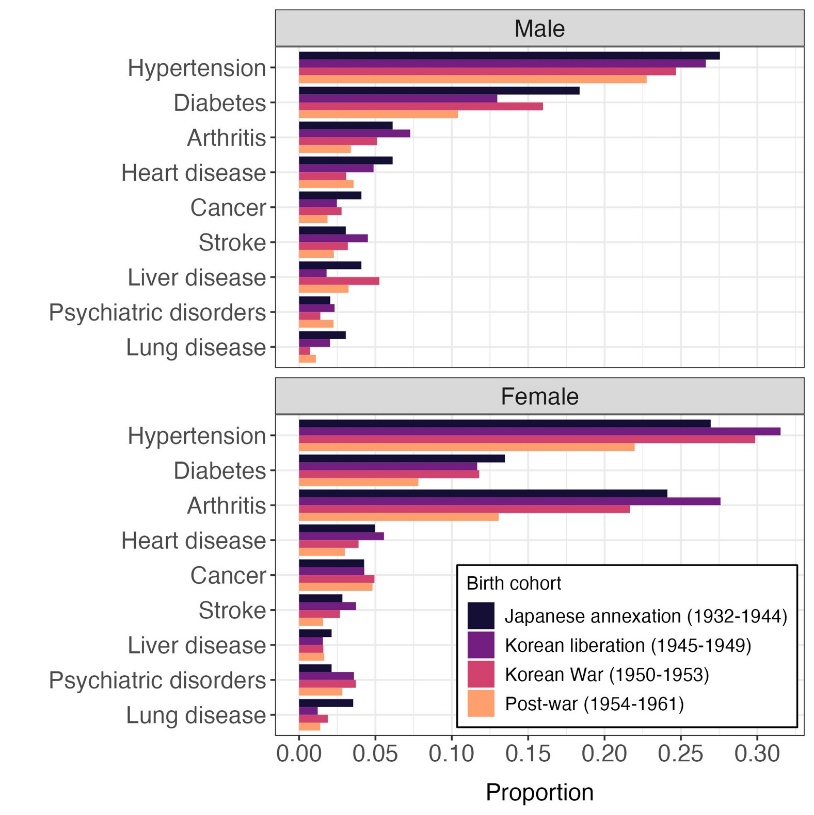 |

**(A)** Distribution of disease proportion across birth cohorts. Only participants aged 62-66 are included in this plot, reflecting the ages in which all cohorts are represented. Data are from the Korean Longitudinal Study of Aging (2006-2020).

**(B)** Distribution of disease proportion across sex and birth cohorts. Only participants aged 62-66 are included in this plot, reflecting the ages in which all cohorts are represented. Data are from the Korean Longitudinal Study of Aging (2006-2020).

**Section 3:** **Models stratified by sex.**

**Supplementary Table 1.** Incidence rate ratios with 95% confidence intervals for disease accumulation across four birth cohorts for **males**, adjusted for age, early-life exposures, and adult characteristics, using data from the Korean Longitudinal Study of Aging (2006-2020)

|  |  | **Model 1** | **Model 2** | **Model 3** | **Model 4** | **Model 5** |
| --- | --- | --- | --- | --- | --- | --- |
| **Intercept** |  | 0.44 (0.40–0.49)^***^ | 0.45 (0.41–0.51)^***^ | 0.45 (0.40–0.51)^***^ | 0.42 (0.36–0.49)^***^ | 0.41 (0.35–0.48)^***^ |
| **Age^a^** |  | 1.07 (1.06–1.07)^***^ | 1.07 (1.06–1.08)^***^ | 1.07 (1.06–1.08)^***^ | 1.07 (1.06–1.08)^***^ | 1.07 (1.06–1.08)^***^ |
| **Birth cohort** | Post-war (1954-1961) | *Ref* | *Ref* | *Ref* | *Ref* | *Ref* |
|  | Japanese annexation (1932-1944) | 1.02 (0.89–1.17) | 0.98 (0.84–1.14) | 0.96 (0.82–1.12) | 0.97 (0.83–1.13) | 0.96 (0.82–1.13) |
|  | Korean liberation (1945-1949) | 1.12 (0.96–1.31) | 1.10 (0.94–1.29) | 1.09 (0.93–1.28) | 1.07 (0.91–1.25) | 1.07 (0.91–1.25) |
|  | Korean War (1950-1953) | 1.09 (0.93–1.29) | 1.06 (0.89–1.26) | 1.05 (0.88–1.25) | 1.02 (0.86–1.21) | 1.02 (0.86–1.22) |
| **Age:Birth cohort interaction** | Age:Post-war (1954-1961) |  | *Ref* | *Ref* | *Ref* | *Ref* |
|  | Age:Japanese annexation (1932-1944) |  | 1.00 (0.98–1.01) | 1.00 (0.98–1.01) | 1.00 (0.98–1.01) | 1.00 (0.98–1.01) |
|  | Age:Korean liberation (1945-1949) |  | 0.99 (0.98–1.00) | 0.99 (0.98–1.00) | 0.99 (0.98–1.00) | 0.99 (0.98–1.00) |
|  | Age:Korean War (1950-1953) |  | 0.99 (0.98–1.01) | 0.99 (0.98–1.01) | 1.00 (0.98–1.01) | 1.00 (0.98–1.01) |
| **Parental death during childhood** | No |  |  | *Ref* |  | *Ref* |
|  | Yes |  |  | 1.21 (1.05–1.40)^**^ |  | 1.17 (1.02–1.35)^**^ |
| **Parental education** | No formal education |  |  | *Ref* |  | *Ref* |
|  | Elementary school |  |  | 1.01 (0.90–1.13) |  | 1.03 (0.92–1.16) |
|  | Middle school or more |  |  | 0.92 (0.79–1.08) |  | 1.01 (0.86–1.19) |
| **Own education** | Middle school or less |  |  |  | *Ref* | *Ref* |
|  | High school |  |  |  | 0.90 (0.81–1.01) | 0.90 (0.80–1.01) |
|  | College/university |  |  |  | 0.79 (0.69–0.91)^**^ | 0.79 (0.68–0.92)^**^ |
| **Place of residence** | Urban |  |  |  | *Ref* | *Ref* |
|  | Rural |  |  |  | 0.91 (0.83–1.00) | 0.92 (0.84–1.01) |
| **Smoking status** | Never smoker |  |  |  | *Ref* | *Ref* |
|  | Ever smoker |  |  |  | 1.14 (1.03–1.26)^*^ | 1.13 (1.03–1.25)^*^ |
| **Obesity status** | Not obese |  |  |  | *Ref* | *Ref* |
|  | Obese |  |  |  | 1.64 (1.47–1.84)^***^ | 1.64 (1.46–1.83)^***^ |
| **MMSE Score** |  |  |  |  |  |  |
| **Random intercept variance** |  | 1.65 | 1.65 | 1.64 | 1.58 | 1.58 |
| **Intraclass Correlation Coefficient (ICC)** |  | 0.73 | 0.73 | 0.73 | 0.73 | 0.72 |

*Note:* Results were computed using Poisson mixed-effects models with repeated measures at level 1 and individuals at level 2. Due to rounding, some confidence intervals appear to include 1.00 but remain statistically significant.

^a^ Age is grand-mean centered at 63.0 years.

^*^p<0.05, ^**^p<0.01, ^***^p<0.001

**Supplementary Table 2.** Incidence rate ratios with 95% confidence intervals for disease accumulation across four birth cohorts for **female**, adjusted for age, early-life exposures, and adult characteristics, using data from the Korean Longitudinal Study of Aging (2006-2020)

|  |  | **Model 1** | **Model 2** | **Model 3** | **Model 4** | **Model 5** |
| --- | --- | --- | --- | --- | --- | --- |
| **Intercept** |  | 0.53 (0.49–0.57)^***^ | 0.56 (0.52–0.61) ^***^ | 0.60 (0.54–0.66)^***^ | 0.61 (0.55–0.68)^***^ | 0.61 (0.55–0.68)^***^ |
| **Age^a^** |  | 1.06 (1.06–1.06)^***^ | 1.07 (1.06–1.08)^***^ | 1.07 (1.06–1.08)^***^ | 1.07 (1.06–1.08)^***^ | 1.07 (1.06–1.08)^***^ |
| **Birth cohort** | Post-war (1954-1961) | *Ref* | *Ref* | *Ref* | *Ref* | *Ref* |
|  | Japanese annexation (1932-1944) | 1.39 (1.25–1.53)^***^ | 1.32 (1.18–1.48)^***^ | 1.27 (1.13–1.43)^***^ | 1.10 (0.98–1.23) | 1.10 (0.98–1.23) |
|  | Korean liberation (1945-1949) | 1.49 (1.33–1.67)^***^ | 1.42 (1.26–1.60)^***^ | 1.38 (1.22–1.56)^***^ | 1.23 (1.09–1.39)^***^ | 1.23 (1.09–1.39)^***^ |
|  | Korean War (1950-1953) | 1.33 (1.18–1.50)^***^ | 1.25 (1.10–1.42)^***^ | 1.22 (1.07–1.39)^**^ | 1.09 (0.96–1.24) | 1.09 (0.96–1.24) |
| **Age:Birth cohort interaction** | Age:Post-war (1954-1961) |  | *Ref* | *Ref* | *Ref* | *Ref* |
|  | Age:Japanese annexation (1932-1944) |  | 0.99 (0.98–1.00)^*^ | 0.99 (0.98–1.00)^*^ | 0.99 (0.98–1.00)^*^ | 0.99 (0.98-–1.00)^*^ |
|  | Age:Korean liberation (1945-1949) |  | 0.99 (0.98–0.99)^**^ | 0.99 (0.98–0.99)^**^ | 0.99 (0.98–1.00)^**^ | 0.99 (0.98–1.00)^**^ |
|  | Age:Korean War (1950-1953) |  | 0.99 (0.98–1.00)^*^ | 0.99 (0.98–1.00)^*^ | 0.99 (0.98–1.00)^*^ | 0.99 (0.98–1.00)^*^ |
| **Parental death during childhood** | No |  |  | *Ref* |  | *Ref* |
|  | Yes |  |  | 1.06 (0.94–1.19) |  | 1.02 (0.92–1.15) |
| **Parental education** | No formal education |  |  | *Ref* |  | *Ref* |
|  | Elementary school |  |  | 0.93 (0.86–1.01) |  | 1.00 (0.92–1.09) |
|  | Middle school or more |  |  | 0.83 (0.74–0.94)^**^ |  | 1.00 (0.88–1.13) |
| **Own education** | Middle school or less |  |  |  | *Ref* | *Ref* |
|  | High school |  |  |  | 0.75 (0.68–0.83)^***^ | 0.75  (0.68–0.83)^***^ |
|  | College/university |  |  |  | 0.56 (0.46–0.67)^***^ | 0.56 (0.46–0.67)^***^ |
| **Place of residence** | Urban |  |  |  | *Ref* | *Ref* |
|  | Rural |  |  |  | 0.91 (0.85–0.98)^*^ | 0.91 (0.85–0.98)^*^ |
| **Smoking status** | Never smoker |  |  |  | *Ref* | *Ref* |
|  | Ever smoker |  |  |  | 1.30 (1.07–1.58)^**^ | 1.30 (1.07–1.58)^**^ |
| **Obesity status** | Not obese |  |  |  | *Ref* | *Ref* |
|  | Obese |  |  |  | 1.68 (1.55–1.81)^***^ | 1.68 (1.55–1.81)^***^ |
| **Random intercept variance** |  | 1.09 | 1.09 | 1.08 | 0.98 | 0.98 |
| **Intraclass Correlation Coefficient (ICC)** |  | 0.54 | 0.54 | 0.54 | 0.54 | 0.54 |

*Note:* Results were computed using Poisson mixed-effects models with repeated measures at level 1 and individuals at level 2. Due to rounding, some confidence intervals appear to include 1.00 but remain statistically significant.

^a^ Age is grand-mean centered at 62.8 years.

^*^p<0.05, ^**^p<0.01, ^***^p<0.001

**Section 4:** **Sensitivity analyses.**

**Supplementary Table 3**. Incidence rate ratios with 95% confidence intervals for disease accumulation across four birth cohorts **for low- and high-mortality diseases with and without psychiatric disorders**, adjusted for sex, age, early-life exposures, and adult characteristics, using data from the Korean Longitudinal Study of Aging (2006-2020)

|  |  | **Low-mortality** | **Low-mortality, with psychiatric disorders** | **High-mortality** | **High-mortality, with psychiatric disorders** |
| --- | --- | --- | --- | --- | --- |
| **Intercept** |  | 0.20 (0.18–0.23)^***^ | 0.23 (0.20–0.26)^***^ | 0.12 (0.10–0.15)^***^ | 0.14 (0.11–0.17)^***^ |
| **Sex** | Male | *Ref* | *Ref* | *Ref* | *Ref* |
|  | Female | 1.60 (1.46–1.76)^***^ | 1.61 (1.47–1.76)^***^ | 0.92 (0.80–1.05) | 0.98 (0.85–1.12) |
| **Age^a^** |  | 1.06 (1.05–1.07)^***^ | 1.06 (1.05–1.07)^***^ | 1.08 (1.08–1.09)^***^ | 1.08 (1.07–1.09)^***^ |
| **Birth cohort** | Post-war (1954-1961) | *Ref* | *Ref* | *Ref* | *Ref* |
|  | Japanese annexation (1932-1944) | 1.26 (1.12–1.41)^***^ | 1.18 (1.06–1.32)^**^ | 1.04 (0.88–1.23) | 0.98 (0.84–1.15) |
|  | Korean liberation (1945-1949) | 1.28 (1.14–1.44)^***^ | 1.25 (1.12–1.40)^***^ | 1.10 (0.93–1.30) | 1.08 (0.92–1.28) |
|  | Korean War (1950-1953) | 1.13 (1.01–1.28)^*^ | 1.10 (0.98–1.24) | 1.06 (0.88–1.26) | 1.02 (0.86–1.22) |
| **Age:Birth cohort interaction** | Age:Post-war (1954-1961) |  | *Ref* | *Ref* | *Ref* |
|  | Age:Japanese annexation (1932-1944) | 0.99 (0.98–1.00) | 1.00 (0.98–1.01) | 0.99 (0.98–1.00) | 0.99 (0.98–1.00) |
|  | Age:Korean liberation (1945-1949) | 0.99 (0.98–1.00)^**^ | 0.99 (0.98–1.00)^*^ | 0.99 (0.97–1.00)^*^ | 0.99 (0.98–1.00)^*^ |
|  | Age:Korean War (1950-1953) | 0.99 (0.98–1.00) | 0.99 (0.98–1.00) | 0.99 (0.97–1.00)^*^ | 0.99 (0.98–1.00)^*^ |
| **Parental death during childhood** | No |  |  | *Ref* |  |
|  | Yes | 1.08 (0.97–1.19) | 1.07 (0.97–1.19) | 1.13 (0.97–1.32) | 1.11 (0.95–1.29) |
| **Parental education** | No formal education |  |  | *Ref* |  |
|  | Elementary school | 0.97 (0.90–1.05) | 0.96 (0.88–1.04) | 1.06 (0.94–1.20) | 1.05 (0.93–1.18) |
|  | Middle school or more | 0.94 (0.84–1.05) | 0.96 (0.86–1.08) | 1.08 (0.91–1.28) | 1.10 (0.93–1.30) |
| **Own education** | Middle school or less |  |  |  | *Ref* |
|  | High school | 0.80 (0.73–0.87)^***^ | 0.78 (0.72–0.85)^***^ | 0.86 (0.75–0.98)^*^ | 0.83 (0.73–0.94)^**^ |
|  | College/university | 0.73 (0.64–0.83)^***^ | 0.70 (0.62–0.80)^***^ | 0.72 (0.59–0.88)^**^ | 0.69 (0.57–0.84)^***^ |
| **Place of residence** | Urban |  |  |  | *Ref* |
|  | Rural | 0.90 (0.84–0.97)^**^ | 0.90 (0.84–0.96)^**^ | 0.92 (0.84–1.01) | 0.91 (0.83–1.00)^*^ |
| **Smoking status** | Never smoker |  |  |  | *Ref* |
|  | Ever smoker | 1.02 (0.93–1.13) | 1.04 (0.95–1.15) | 1.34 (1.16–1.55)^***^ | 1.37 (1.19–1.58)^***^ |
| **Obesity status** | Not obese |  |  |  | *Ref* |
|  | Obese | 1.82 (1.69–1.96) | 1.76 (1.64–1.90) | 1.59 (1.42–1.78)^***^ | 1.53 (1.37–1.71)^***^ |
| **Random intercept variance** |  | 1.42 | 1.41 | 3.35 | 3.24 |
| **Intraclass Correlation Coefficient (ICC)** |  | 0.65 | 0.65 | 0.82 | 0.82 |

*Note:* Results were computed using Poisson mixed-effects models with repeated measures at level 1 and individuals at level 2. Due to rounding, some confidence intervals appear to include 1.00 but remain statistically significant. Results are from fully adjusted models.

^a^ Age is grand-mean centered at 62.9 years.

^*^p<0.05, ^**^p<0.01, ^***^p<0.001

**Supplementary Table 4**. Incidence rate ratios with 95% confidence intervals for disease accumulation across four birth cohorts **excluding hypertension**, adjusted for sex, age, early-life exposures, and adult characteristics, using data from the Korean Longitudinal Study of Aging (2006-2020)

|  |  | **Model 1** | **Model 2** | **Model 3** | **Model 4** | **Model 5** |
| --- | --- | --- | --- | --- | --- | --- |
| **Intercept** |  | 0.18 (0.17–0.20)^***^ | 0.19 (0.17–0.21)^***^ | 0.20 (0.18–0.23)^***^ | 0.20 (0.17–0.23)^***^ | 0.20 (0.17–0.23)^***^ |
| **Sex** | Male | *Ref* | *Ref* | *Ref* | *Ref* | *Ref* |
|  | Female | 1.49 (1.38–1.61)^***^ | 1.49 (1.38–1.61)^***^ | 1.50 (1.38–1.62)^***^ | 1.53 (1.37–1.70)^***^ | 1.53 (1.38–1.70)^***^ |
| **Age^a^** |  | 1.07 (1.06–1.07)^***^ | 1.08 (1.07–1.08)^***^ | 1.08 (1.07–1.08)^***^ | 1.08 (1.07–1.08)^***^ | 1.08 (1.07–1.08)^***^ |
| **Birth cohort** | Post-war (1954-1961) | *Ref* | *Ref* | *Ref* | *Ref* | *Ref* |
|  | Japanese annexation (1932-1944) | 1.40 (1.26–1.56)^***^ | 1.33 (1.18–1.50)^***^ | 1.27 (1.13–1.44)^***^ | 1.16 (1.02–1.31)^*^ | 1.15 (1.02–1.30)^*^ |
|  | Korean liberation (1945-1949) | 1.45 (1.28–1.64)^***^ | 1.39 (1.22–1.58)^***^ | 1.34 (1.18–1.53)^***^ | 1.23 (1.08–1.40)^**^ | 1.23 (1.08–1.40)^**^ |
|  | Korean War (1950-1953) | 1.30 (1.14–1.48)^***^ | 1.22 (1.07–1.40)^**^ | 1.20 (1.04–1.37)^*^ | 1.10 (0.96–1.26) | 1.10 (0.96–1.26) |
| **Age:Birth cohort interaction** | Age:Post-war (1954-1961) |  | *Ref* | *Ref* | *Ref* | *Ref* |
|  | Age:Japanese annexation (1932-1944) |  | 0.99 (0.98–1.00) | 0.99 (0.98–1.00) | 0.99 (0.98–1.00) | 0.99 (0.98–1.00) |
|  | Age:Korean liberation (1945-1949) |  | 0.99 (0.98–1.00)^**^ | 0.99 (0.98–1.00)^**^ | 0.99 (0.98–1.00)^**^ | 0.99 (0.98–1.00)^**^ |
|  | Age:Korean War (1950-1953) |  | 0.99 (0.98–1.00)^*^ | 0.99 (0.98–1.00)^*^ | 0.99 (0.98–1.00)^*^ | 0.99 (0.98–1.00)^*^ |
| **Parental death during childhood** | No |  |  | *Ref* |  | *Ref* |
|  | Yes |  |  | 1.18 (1.05–1.33)^**^ |  | 1.13 (1.01–1.28)^*^ |
| **Parental education** | No formal education |  |  | *Ref* |  | *Ref* |
|  | Elementary school |  |  | 0.91 (0.83–1.00)^*^ |  | 0.99 (0.91–1.09) |
|  | Middle school or more |  |  | 0.85 (0.75–0.96)^**^ |  | 1.02 (0.90–1.17) |
| **Own education** | Middle school or less |  |  |  | *Ref* | *Ref* |
|  | High school |  |  |  | 0.74 (0.67–0.81)^***^ | 0.74 (0.67–0.82)^***^ |
|  | College/university |  |  |  | 0.60 (0.52–0.69)^***^ | 0.60 (0.51–0.70)^***^ |
| **Place of residence** | Urban |  |  |  | *Ref* | *Ref* |
|  | Rural |  |  |  | 0.94 (0.87–1.01) | 0.94 (0.87–1.01) |
| **Smoking status** | Never smoker |  |  |  | *Ref* | *Ref* |
|  | Ever smoker |  |  |  | 1.30 (1.16–1.45)^***^ | 1.29 (1.15–1.44)^***^ |
| **Obesity status** | Not obese |  |  |  | *Ref* | *Ref* |
|  | Obese |  |  |  | 1.58 (1.45–1.72)^***^ | 1.58 (1.45–1.72)^***^ |
| **Random intercept variance** |  | 2.13 | 2.13 | 2.12 | 2.03 | 2.02 |
| **Intraclass Correlation Coefficient (ICC)** |  | 0.76 | 0.76 | 0.76 | 0.76 | 0.75 |

*Note:* Results were computed using Poisson mixed-effects models with repeated measures at level 1 and individuals at level 2. Due to rounding, some confidence intervals appear to include 1.00 but remain statistically significant.

^a^ Age is grand-mean centered at 62.9 years.

^*^p<0.05, ^**^p<0.01, ^***^p<0.001

**Supplementary Table 5**. Incidence rate ratios with 95% confidence intervals for disease accumulation across four birth cohorts **excluding arthritis**, adjusted for sex, age, early-life exposures, and adult characteristics, using data from the Korean Longitudinal Study of Aging (2006-2020)

|  |  | **Model 1** | **Model 2** | **Model 3** | **Model 4** | **Model 5** |
| --- | --- | --- | --- | --- | --- | --- |
| **Intercept** |  | 0.37 (0.35–0.40)^***^ | 0.39 (0.36–0.43)^***^ | 0.39 (0.35–0.43)^***^ | 0.37 (0.33–0.42)^***^ | 0.36 (0.32–0.41)^***^ |
| **Sex** | Male | *Ref* | *Ref* | *Ref* | *Ref* | *Ref* |
|  | Female | 1.03 (0.97–1.11) | 1.03 (0.97–1.11) | 1.04 (0.97–1.11) | 1.05 (0.96–1.15) | 1.05 (0.96–1.15) |
| **Age^a^** |  | 1.06 (1.06–1.07)^***^ | 1.07 (1.07–1.08)^***^ | 1.07 (1.07–1.08)^***^ | 1.07 (1.07–1.08)^***^ | 1.07 (1.07–1.08)^***^ |
| **Birth cohort** | Post-war (1954-1961) | *Ref* | *Ref* | *Ref* | *Ref* | *Ref* |
|  | Japanese annexation (1932-1944) | 1.09 (1.00–1.20) | 1.05 (0.95–1.17) | 1.04 (0.94–1.16) | 0.99 (0.89–1.11) | 1.00 (0.89–1.11) |
|  | Korean liberation (1945-1949) | 1.21 (1.09–1.35)^***^ | 1.17 (1.05–1.31)^**^ | 1.16 (1.04–1.30)^**^ | 1.11 (0.99–1.23) | 1.11 (0.99–1.24) |
|  | Korean War (1950-1953) | 1.16 (1.03–1.29)^*^ | 1.10 (0.98–1.24) | 1.09 (0.97–1.23) | 1.03 (0.92–1.16) | 1.04 (0.92–1.16) |
| **Age:Birth cohort interaction** | Age:Post-war (1954-1961) |  | *Ref* | *Ref* | *Ref* | *Ref* |
|  | Age:Japanese annexation (1932-1944) |  | 0.99 (0.98–1.00)^*^ | 0.99 (0.98–1.00)^*^ | 0.99 (0.98–1.00) | 0.99 (0.98–1.00) |
|  | Age:Korean liberation (1945-1949) |  | 0.99 (0.98–1.00)^**^ | 0.99 (0.98–1.00)^**^ | 0.99 (0.98–1.00)^**^ | 0.99 (0.98–1.00)^**^ |
|  | Age:Korean War (1950-1953) |  | 0.99 (0.98–1.00)^*^ | 0.99 (0.98–1.00)^*^ | 0.99 (0.98–1.00)^*^ | 0.99 (0.98–1.00)^*^ |
| **Parental death during childhood** | No |  |  | *Ref* |  | *Ref* |
|  | Yes |  |  | 1.10 (0.99–1.22) |  | 1.07 (0.96–1.18) |
| **Parental education** | No formal education |  |  | *Ref* |  | *Ref* |
|  | Elementary school |  |  | 1.03 (0.95–1.11) |  | 1.06 (0.97–1.14) |
|  | Middle school or more |  |  | 0.93 (0.84–1.04) |  | 1.02 (0.91–1.14) |
| **Own education** | Middle school or less |  |  |  | *Ref* | *Ref* |
|  | High school |  |  |  | 0.88 (0.81–0.95)^**^ | 0.87 (0.80–0.95)^**^ |
|  | College/university |  |  |  | 0.78 (0.69–0.88)^***^ | 0.77 (0.68–0.88)^***^ |
| **Place of residence** | Urban |  |  |  | *Ref* | *Ref* |
|  | Rural |  |  |  | 0.89 (0.84–0.95)^***^ | 0.89 (0.84–0.95)^***^ |
| **Smoking status** | Never smoker |  |  |  | *Ref* | *Ref* |
|  | Ever smoker |  |  |  | 1.17 (1.06–1.29)^**^ | 1.17 (1.06–1.28)^**^ |
| **Obesity status** | Not obese |  |  |  | *Ref* | *Ref* |
|  | Obese |  |  |  | 1.70 (1.58–1.84)^***^ | 1.70 (1.58–1.83)^***^ |
| **Random intercept variance** |  | 1.69 | 1.68 | 1.68 | 1.60 | 1.60 |
| **Intraclass Correlation Coefficient (ICC)** |  | 0.73 | 0.73 | 0.73 | 0.72 | 0.72 |

*Note:* Results were computed using Poisson mixed-effects models with repeated measures at level 1 and individuals at level 2. Due to rounding, some confidence intervals appear to include 1.00 but remain statistically significant.

^a^ Age is grand-mean centered at 62.9 years.

^*^p<0.05, ^**^p<0.01, ^***^p<0.001

**Supplementary Table 6**. Incidence rate ratios with 95% confidence intervals for disease accumulation across four birth cohorts **excluding hypertension and arthritis**, adjusted for sex, age, early-life exposures, and adult characteristics, using data from the Korean Longitudinal Study of Aging (2006-2020)

|  |  | **Model 1** | **Model 2** | **Model 3** | **Model 4** | **Model 5** |
| --- | --- | --- | --- | --- | --- | --- |
| **Intercept** |  | 0.15 (0.13–0.17)^***^ | 0.16 (0.14–0.18)^***^ | 0.16 (0.14–0.18)^***^ | 0.14 (0.12–0.17)^***^ | 0.14 (0.11–0.17)^***^ |
| **Sex** | Male | *Ref* | *Ref* | *Ref* | *Ref* | *Ref* |
|  | Female | 0.89 (0.80–0.98)^*^ | 0.89 (0.80–0.98)^*^ | 0.89 (0.81–0.99)^*^ | 0.98 (0.86–1.13) | 0.98 (0.85–1.12) |
| **Age^a^** |  | 1.07 (1.07–1.08)^***^ | 1.08 (1.07–1.09)^***^ | 1.08 (1.07–1.09)^***^ | 1.08 (1.07–1.09)^***^ | 1.08 (1.07–1.09)^***^ |
| **Birth cohort** | Post-war (1954-1961) | *Ref* | *Ref* | *Ref* | *Ref* | *Ref* |
|  | Japanese annexation (1932-1944) | 1.10 (0.96–1.27) | 1.05 (0.91–1.23) | 1.04 (0.89–1.22) | 0.98 (0.83–1.14) | 0.98 (0.84–1.15) |
|  | Korean liberation (1945-1949) | 1.20 (1.03–1.41)^*^ | 1.16 (0.98–1.36) | 1.15 (0.97–1.35) | 1.08 (0.92–1.27) | 1.08 (0.92–1.28) |
|  | Korean War (1950-1953) | 1.15 (0.97–1.36) | 1.09 (0.92–1.30) | 1.09 (0.91–1.30) | 1.02 (0.85–1.21) | 1.02 (0.86–1.22) |
| **Age:Birth cohort interaction** | Age:Post-war (1954-1961) |  | *Ref* | *Ref* | *Ref* | *Ref* |
|  | Age:Japanese annexation (1932-1944) |  | 0.99 (0.98–1.00) | 0.99 (0.98–1.00) | 0.99 (0.98–1.00) | 0.99 (0.98–1.00) |
|  | Age:Korean liberation (1945-1949) |  | 0.99 (0.98–1.00)^*^ | 0.99 (0.98–1.00)^*^ | 0.99 (0.98–1.00)^*^ | 0.99 (0.98–1.00)^*^ |
|  | Age:Korean War (1950-1953) |  | 0.99 (0.98–1.00)^*^ | 0.99 (0.98–1.00)^*^ | 0.99 (0.98–1.00)^*^ | 0.99 (0.98–1.00)^*^ |
| **Parental death during childhood** | No |  |  | *Ref* |  | *Ref* |
|  | Yes |  |  | 1.15 (0.98–1.34) |  | 1.11 (0.95–1.29) |
| **Parental education** | No formal education |  |  | *Ref* |  | *Ref* |
|  | Elementary school |  |  | 0.99 (0.88–1.11) |  | 1.05 (0.93–1.18) |
|  | Middle school or more |  |  | 0.97 (0.82–1.13) |  | 1.10 (0.93–1.30) |
| **Own education** | Middle school or less |  |  |  | *Ref* | *Ref* |
|  | High school |  |  |  | 0.85 (0.75–0.96)^**^ | 0.83 (0.73–0.94)^**^ |
|  | College/university |  |  |  | 0.71 (0.59–0.86)^***^ | 0.69 (0.57–0.84)^***^ |
| **Place of residence** | Urban |  |  |  | *Ref* | *Ref* |
|  | Rural |  |  |  | 0.91 (0.83–1.00)^*^ | 0.91 (0.83–1.00)^*^ |
| **Smoking status** | Never smoker |  |  |  | *Ref* | *Ref* |
|  | Ever smoker |  |  |  | 1.38 (1.20–1.58)^***^ | 1.37 (1.19–1.58)^***^ |
| **Obesity status** | Not obese |  |  |  | *Ref* | *Ref* |
|  | Obese |  |  |  | 1.53 (1.37–1.72)^***^ | 1.53 (1.37–1.71)^***^ |
| **Random intercept variance** |  | 3.32 | 3.32 | 3.32 | 3.24 | 3.24 |
| **Intraclass Correlation Coefficient (ICC)** |  | 0.83 | 0.83 | 0.83 | 0.82 | 0.82 |

*Note:* Results were computed using Poisson mixed-effects models with repeated measures at level 1 and individuals at level 2. Due to rounding, some confidence intervals appear to include 1.00 but remain statistically significant.

^a^ Age is grand-mean centered at 62.9 years.

^*^p<0.05, ^**^p<0.01, ^***^p<0.001

**Supplementary Table 7**. Incidence rate ratios with 95% confidence intervals for disease accumulation across four birth cohorts **for age range 50-70 years**, adjusted for sex, age, early-life exposures, and adult characteristics, using data from the Korean Longitudinal Study of Aging (2006-2020)

|  |  | **Model 1** | **Model 2** | **Model 3** | **Model 4** | **Model 5** |
| --- | --- | --- | --- | --- | --- | --- |
| **Intercept** |  | 0.35 (0.33–0.38)^***^ | 0.36 (0.33–0.38)^***^ | 0.37 (0.34–0.41)^***^ | 0.37 (0.33–0.41)^***^ | 0.36 (0.32–0.41)^***^ |
| **Sex** | Male | *Ref* | *Ref* | *Ref* | *Ref* | *Ref* |
|  | Female | 1.34 (1.25–1.43)^***^ | 1.34 (1.25–1.43)^***^ | 1.34 (1.25–1.43)^***^ | 1.34 (1.22–1.47)^***^ | 1.34 (1.22–1.47)^***^ |
| **Age^a^** |  | 1.07 (1.06–1.07)^***^ | 1.07 (1.06–1.08)^***^ | 1.07 (1.06–1.08)^***^ | 1.07 (1.06–1.08)^***^ | 1.07 (1.06–1.08)^***^ |
| **Birth cohort** | Post-war (1954-1961) | *Ref* | *Ref* | *Ref* | *Ref* | *Ref* |
|  | Japanese annexation (1932-1944) | 1.28 (1.17–1.40)^***^ | 1.21 (1.08–1.35)^***^ | 1.17 (1.04–1.30)^**^ | 1.07 (0.95–1.19) | 1.06 (0.95–1.19) |
|  | Korean liberation (1945-1949) | 1.33 (1.21–1.47)^***^ | 1.31 (1.18–1.45)^***^ | 1.27 ^***^ (1.15–1.41)^***^ | 1.18 (1.07–1.30)^**^ | 1.18 (1.06–1.30)^**^ |
|  | Korean War (1950-1953) | 1.21 (1.09–1.34)^***^ | 1.18 (1.06–1.32)^**^ | 1.16 (1.04–1.29)^**^ | 1.07 (0.97–1.20) | 1.08 (0.97–1.20) |
| **Age:Birth cohort interaction** | Age:Post-war (1954-1961) |  | *Ref* | *Ref* | *Ref* | *Ref* |
|  | Age:Japanese annexation (1932-1944) |  | 1.01 (0.99–1.02) | 1.01 (0.99–1.02) | 1.01 (0.99–1.02) | 1.01 (0.99–1.02) |
|  | Age:Korean liberation (1945-1949) |  | 1.00 (0.99–1.01) | 1.00 (0.99–1.01) | 1.00 (0.99–1.01) | 1.00 (0.99–1.01) |
|  | Age:Korean War (1950-1953) |  | 0.99 (0.98–1.00)^*^ | 0.99 (0.98–1.00)^*^ | 0.99 (0.98–1.00)^*^ | 0.99 (0.98–1.00)^*^ |
| **Parental death during childhood** | No |  |  | *Ref* |  | *Ref* |
|  | Yes |  |  | 1.14 (1.03–1.27)^*^ |  | 1.10 (0.99–1.22) |
| **Parental education** | No formal education |  |  | *Ref* |  | *Ref* |
|  | Elementary school |  |  | 0.95 (0.88–1.03) |  | 1.02 (0.94–1.11) |
|  | Middle school or more |  |  | 0.85 (0.77–0.95)^**^ |  | 1.00 (0.89–1.11) |
| **Own education** | Middle school or less |  |  |  | *Ref* | *Ref* |
|  | High school |  |  |  | 0.78 (0.72–0.84)^***^ | 0.78 (0.71–0.84)^***^ |
|  | College/university |  |  |  | 0.66 (0.59–0.75)^***^ | 0.67 (0.59–0.76)^***^ |
| **Place of residence** | Urban |  |  |  | *Ref* | *Ref* |
|  | Rural |  |  |  | 0.91 (0.85–0.98)^*^ | 0.92 (0.86–0.98)^*^ |
| **Smoking status** | Never smoker |  |  |  | *Ref* | *Ref* |
|  | Ever smoker |  |  |  | 1.20 (1.09–1.32)^***^ | 1.20 (1.09–1.32)^***^ |
| **Obesity status** | Not obese |  |  |  | *Ref* | *Ref* |
|  | Obese |  |  |  | 1.70 (1.58–1.83)^***^ | 1.70 (1.58–1.83)^***^ |
| **Random intercept variance** |  | 1.46 | 1.46 | 1.45 | 1.35 | 1.35 |
| **Intraclass Correlation Coefficient (ICC)** |  | 0.71 | 0.71 | 0.70 | 0.69 | 0.69 |

*Note:* Results were computed using Poisson mixed-effects models with repeated measures at level 1 and individuals at level 2. Due to rounding, some confidence intervals appear to include 1.00 but remain statistically significant.

^a^ Age is grand-mean centered at 60.5 years.

^*^p<0.05, ^**^p<0.01, ^***^p<0.001

**Supplementary Table 8**. Incidence rate ratios with 95% confidence intervals for disease accumulation across four birth cohorts **for age range 55-70 years**, adjusted for sex, age, early-life exposures, and adult characteristics, using data from the Korean Longitudinal Study of Aging (2006-2020)

|  |  | **Model 1** | **Model 2** | **Model 3** | **Model 4** | **Model 5** |
| --- | --- | --- | --- | --- | --- | --- |
| **Intercept** |  | 0.45 (0.42–0.49)^***^ | 0.45 (0.42–0.48)^***^ | 0.46 (0.43–0.51)^***^ | 0.45 (0.40–0.51)^***^ | 0.44 (0.40–0.50)^***^ |
| **Sex** | Male | *Ref* | *Ref* | *Ref* | *Ref* | *Ref* |
|  | Female | 1.34 (1.26–1.43)^***^ | 1.34 (1.26–1.43)^***^ | 1.34 (1.26–1.44)^***^ | 1.36 (1.25–1.49)^***^ | 1.36 (1.25–1.49)^***^ |
| **Age^a^** |  | 1.06 (1.06–1.07)^***^ | 1.06 (1.05–1.07)^***^ | 1.06 (1.05–1.07)^***^ | 1.06 (1.05–1.07)^***^ | 1.06 (1.05–1.07)^***^ |
| **Birth cohort** | Post-war (1954-1961) | *Ref* | *Ref* | *Ref* | *Ref* | *Ref* |
|  | Japanese annexation (1932-1944) | 1.16 (1.06–1.27)^***^ | 1.12 (1.00–1.24)^*^ | 1.08 (0.97–1.21) | 1.00 (0.90–1.12) | 1.00 (0.90–1.12) |
|  | Korean liberation (1945-1949) | 1.20 (1.10–1.32)^***^ | 1.21 (1.10–1.33)^***^ | 1.18 (1.07–1.30)^***^ | 1.11(1.01–1.22)^*^ | 1.11 (1.00–1.22)^*^ |
|  | Korean War (1950-1953) | 1.12 (1.01–1.24)^*^ | 1.13 (1.01–1.25)^*^ | 1.11 (1.00–1.23) | 1.04 (0.94–1.15) | 1.04 (0.94–1.15) |
| **Age:Birth cohort interaction** | Age:Post-war (1954-1961) |  | *Ref* | *Ref* | *Ref* | *Ref* |
|  | Age:Japanese annexation (1932-1944) |  | 1.02 (1.00–1.03) | 1.02 (1.00–1.03) | 1.02 (1.00–1.04) | 1.02 (1.00–1.04) |
|  | Age:Korean liberation (1945-1949) |  | 1.01 (1.00–1.02) | 1.01 (1.00–1.02) | 1.01 (1.00–1.02) | 1.01 (1.00–1.02) |
|  | Age:Korean War (1950-1953) |  | 1.00 (0.99–1.01) | 1.00 (0.99–1.01) | 1.00 (0.99–1.01) | 1.00 (0.99–1.01) |
| **Parental death during childhood** | No |  |  | *Ref* |  | *Ref* |
|  | Yes |  |  | 1.13 (1.02–1.24)^*^ |  | 1.09 (0.99–1.20) |
| **Parental education** | No formal education |  |  | *Ref* |  | *Ref* |
|  | Elementary school |  |  | 0.96 (0.89–1.04) |  | 1.02 (0.95–1.10) |
|  | Middle school or more |  |  | 0.87 (0.79–0.96)^**^ |  | 1.00 (0.90–1.11) |
| **Own education** | Middle school or less |  |  |  | *Ref* | *Ref* |
|  | High school |  |  |  | 0.80 (0.74–0.86)^***^ | 0.80 (0.74–0.86)^***^ |
|  | College/university |  |  |  | 0.68 (0.60–0.76)^***^ | 0.68 (0.60–0.77)^***^ |
| **Place of residence** | Urban |  |  |  | *Ref* | *Ref* |
|  | Rural |  |  |  | 0.89 (0.84–0.96)^**^ | 0.90 (0.84–0.96)^**^ |
| **Smoking status** | Never smoker |  |  |  | *Ref* | *Ref* |
|  | Ever smoker |  |  |  | 1.21 (1.10–1.33)^***^ | 1.21 (1.10–1.33)^***^ |
| **Obesity status** | Not obese |  |  |  | *Ref* | *Ref* |
|  | Obese |  |  |  | 1.65 (1.53–1.76)^***^ | 1.64 (1.53–1.76)^***^ |
| **Random intercept variance** |  | 1.23 | 1.23 | 1.22 | 1.13 | 1.13 |
| **Intraclass Correlation Coefficient (ICC)** |  | 0.67 | 0.67 | 0.67 | 0.65 | 0.65 |

*Note:* Results were computed using Poisson mixed-effects models with repeated measures at level 1 and individuals at level 2. Due to rounding, some confidence intervals appear to include 1.00 but remain statistically significant.

^a^ Age is grand-mean centered at 62.2 years.

^*^p<0.05, ^**^p<0.01, ^***^p<0.001

**Supplementary Table 9**. Incidence rate ratios with 95% confidence intervals for disease accumulation across four birth cohorts **for age range 55-75 years**, adjusted for sex, age, early-life exposures, and adult characteristics, using data from the Korean Longitudinal Study of Aging (2006-2020)

|  |  | **Model 1** | **Model 2** | **Model 3** | **Model 4** | **Model 5** |
| --- | --- | --- | --- | --- | --- | --- |
| **Intercept** |  | 0.52 (0.48–0.55)^***^ | 0.52 (0.48–0.57)^***^ | 0.54 (0.49–0.59)^***^ | 0.52 (0.47–0.58)^***^ | 0.52 (0.46–0.58)^***^ |
| **Sex** | Male | *Ref* | *Ref* | *Ref* | *Ref* | *Ref* |
|  | Female | 1.36 (1.28–1.44)^***^ | 1.36 (1.28–1.44)^***^ | 1.36 (1.28–1.44)^***^ | 1.35 (1.25–1.46)^***^ | 1.35 (1.25–1.46)^***^ |
| **Age^a^** |  | 1.06 (1.05–1.06)^***^ | 1.06 (1.05–1.07)^***^ | 1.06 (1.05–1.07)^***^ | 1.06 (1.05–1.07)^***^ | 1.06 (1.05–1.07)^***^ |
| **Birth cohort** | Post-war (1954-1961) | *Ref* | *Ref* | *Ref* | *Ref* | *Ref* |
|  | Japanese annexation (1932-1944) | 1.12 (1.03–1.21)^**^ | 1.09 (1.00–1.20) | 1.07 (0.97–1.17) | 1.01 (0.92–1.11) | 1.01 (0.92–1.10) |
|  | Korean liberation (1945-1949) | 1.20 (1.09–1.31)^***^ | 1.19 (1.08–1.31)^***^ | 1.17 (1.06–1.28)^**^ | 1.11 (1.01–1.21)^*^ | 1.10 (1.01–1.21)^*^ |
|  | Korean War (1950-1953) | 1.13 (1.03–1.24)^*^ | 1.12 (1.01–1.24)^*^ | 1.11 (1.00–1.22) | 1.05 (0.95–1.15) | 1.05 (0.95–1.15) |
| **Age:Birth cohort interaction** | Age:Post-war (1954-1961) |  | *Ref* | *Ref* | *Ref* | *Ref* |
|  | Age:Japanese annexation (1932-1944) |  | 1.00 (0.99–1.01) | 1.00 (0.99–1.01) | 1.00 (0.99–1.01) | 1.00 (0.99–1.01) |
|  | Age:Korean liberation (1945-1949) |  | 1.00 (0.99–1.01) | 1.00 (0.99–1.01) | 1.00 (0.99–1.01) | 1.00 (0.99–1.01) |
|  | Age:Korean War (1950-1953) |  | 1.00 (0.99–1.01) | 1.00 (0.99–1.01) | 1.00 (0.99–1.01) | 1.00 (0.99–1.01) |
| **Parental death during childhood** | No |  |  | *Ref* |  | *Ref* |
|  | Yes |  |  | 1.12 (1.03–1.22)^*^ |  | 1.08 (1.00–1.18) |
| **Parental education** | No formal education |  |  | *Ref* |  | *Ref* |
|  | Elementary school |  |  | 0.97 (0.91–1.04) |  | 1.02 (0.95–1.08) |
|  | Middle school or more |  |  | 0.88 (0.81–0.97)^**^ |  | 0.99 (0.90–1.09) |
| **Own education** | Middle school or less |  |  |  | *Ref* | *Ref* |
|  | High school |  |  |  | 0.83 (0.78–0.89)^***^ | 0.83 (0.78–0.90)^***^ |
|  | College/university |  |  |  | 0.72 (0.65–0.80)^***^ | 0.72 (0.65–0.81)^***^ |
| **Place of residence** | Urban |  |  |  | *Ref* | *Ref* |
|  | Rural |  |  |  | 0.90 (0.85–0.95)^***^ | 0.90 (0.85–0.95)^***^ |
| **Smoking status** | Never smoker |  |  |  | *Ref* | *Ref* |
|  | Ever smoker |  |  |  | 1.16 (1.07–1.26)^***^ | 1.16 (1.07–1.26)^***^ |
| **Obesity status** | Not obese |  |  |  | *Ref* | *Ref* |
|  | Obese |  |  |  | 1.64 (1.54–1.74)^***^ | 1.64 (1.54–1.74)^***^ |
| **Random intercept variance** |  | 1.13 | 1.13 | 1.13 | 1.05 | 1.05 |
| **Intraclass Correlation Coefficient (ICC)** |  | 0.67 | 0.67 | 0.67 | 0.65 | 0.65 |

*Note:* Results were computed using Poisson mixed-effects models with repeated measures at level 1 and individuals at level 2. Due to rounding, some confidence intervals appear to include 1.00 but remain statistically significant.

^a^ Age is grand-mean centered at 64.6 years.

^*^p<0.05, ^**^p<0.01, ^***^p<0.001

**Supplementary Table 10**. Incidence rate ratios with 95% confidence intervals for disease accumulation across four birth cohorts **including Mini-Mental State Examination (MMSE) score**, adjusted for sex, age, early-life exposures, and adult characteristics, using data from the Korean Longitudinal Study of Aging (2006-2020)

|  |  | **Model 1** | **Model 2** | **Model 3** | **Model 4** | **Model 5** |
| --- | --- | --- | --- | --- | --- | --- |
| **Intercept** |  | 0.95 (0.76–1.18) | 1.00 (0.80–1.25) | 0.98 (0.78–1.23) | 0.85 (0.68–1.07) | 0.84 (0.67–1.06) |
| **Sex** | Male | *Ref* | *Ref* | *Ref* | *Ref* | *Ref* |
|  | Female | 1.30 (1.22–1.38)^***^ | 1.30 (1.22–1.38)^***^ | 1.30 (1.23–1.39)^***^ | 1.30 (1.20–1.41)^***^ | 1.31 (1.20–1.41)^***^ |
| **Age^a^** |  | 1.06 (1.06–1.06)^***^ | 1.07 (1.06–1.08)^***^ | 1.07 (1.06–1.08)^***^ | 1.07 (1.06–1.08)^***^ | 1.07 (1.06–1.08)^***^ |
| **Birth cohort** | Post-war (1954-1961) | *Ref* | *Ref* | *Ref* | *Ref* | *Ref* |
|  | Japanese annexation (1932-1944) | 1.12 (1.03–1.22)^**^ | 1.08 (0.98–1.19) | 1.06 (0.96–1.17) | 1.01 (0.92–1.12) | 1.01 (0.92–1.12) |
|  | Korean liberation (1945-1949) | 1.29 (1.17–1.42)^***^ | 1.24 (1.13–1.37)^***^ | 1.22 (1.11–1.35)^***^ | 1.16 (1.05–1.28)^**^ | 1.16 (1.05–1.28)^**^ |
|  | Korean War (1950-1953) | 1.22 (1.10–1.35)^***^ | 1.16 (1.04–1.28)^**^ | 1.15 (1.03–1.27)^*^ | 1.08 (0.98–1.20) | 1.08 (0.98–1.20) |
| **Age:Birth cohort interaction** | Age:Post-war (1954-1961) |  | *Ref* | *Ref* | *Ref* | *Ref* |
|  | Age:Japanese annexation (1932-1944) |  | 0.99 (0.98–1.00)^*^ | 0.99 (0.98–1.00)^*^ | 0.99 (0.98–1.00)^*^ | 0.99 (0.98–1.00)^*^ |
|  | Age:Korean liberation (1945-1949) |  | 0.99 (0.98–0.99)^***^ | 0.99 (0.98–0.99)^***^ | 0.99 (0.98–0.99)^***^ | 0.99 (0.98–0.99)^***^ |
|  | Age:Korean War (1950-1953) |  | 0.99 (0.98–1.00)^*^ | 0.99 (0.98–1.00)^*^ | 0.99 (0.98–1.00)^*^ | 0.99 (0.98–1.00)^*^ |
| **Parental death during childhood** | No |  |  | *Ref* |  | *Ref* |
|  | Yes |  |  | 1.12 (1.02–1.22)^*^ |  | 1.08 (0.99–1.19) |
| **Parental education** | No formal education |  |  | *Ref* |  | *Ref* |
|  | Elementary school |  |  | 1.00 (0.93–1.07) |  | 1.03 (0.96–1.10) |
|  | Middle school or more |  |  | 0.91 (0.83–1.00) |  | 1.01 (0.91–1.11) |
| **Own education** | Middle school or less |  |  |  | *Ref* | *Ref* |
|  | High school |  |  |  | 0.85 (0.79–0.92)^***^ | 0.85 (0.79–0.92)^***^ |
|  | College/university |  |  |  | 0.75 (0.67–0.83)^***^ | 0.75 (0.66–0.84)^***^ |
| **Place of residence** | Urban |  |  |  | *Ref* | *Ref* |
|  | Rural |  |  |  | 0.91 (0.86–0.96)^***^ | 0.91 (0.86–0.96)^***^ |
| **Smoking status** | Never smoker |  |  |  | *Ref* | *Ref* |
|  | Ever smoker |  |  |  | 1.15 (1.06–1.25)^***^ | 1.15 (1.06–1.25)^**^ |
| **Obesity status** | Not obese |  |  |  | *Ref* | *Ref* |
|  | Obese |  |  |  | 1.70 (1.59–1.81)^***^ | 1.69 (1.59–1.81)^***^ |
| **MMSE Score** |  | 0.97 (0.96–0.98)^***^ | 0.97 (0.96–0.98)^***^ | 0.97 (0.96–0.98)^***^ | 0.98 (0.97–0.98)^***^ | 0.98 (0.97–0.98)^***^ |
| **Random intercept variance** |  | 1.29 | 1.29 | 1.29 | 1.20 | 1.20 |
| **Intraclass Correlation Coefficient (ICC)** |  | 0.70 | 0.70 | 0.70 | 0.68 | 0.68 |

*Note:* MMSE: Mini-Mental State Examination. Results were computed using Poisson mixed-effects models with repeated measures at level 1 and individuals at level 2. Due to rounding, some confidence intervals appear to include 1.00 but remain statistically significant.

^a^ Age is grand-mean centered at 62.9 years.

^*^p<0.05, ^**^p<0.01, ^***^p<0.001

**Supplementary Table 11**. Incidence rate ratios with 95% confidence intervals for disease accumulation across four birth cohorts **excluding obesity status**, adjusted for sex, age, and adult characteristics, using data from the Korean Longitudinal Study of Aging (2006-2020)

|  |  | **Model 4** | **Model 5** |
| --- | --- | --- | --- |
| **Intercept** |  | 0.50 (0.45–0.56)^***^ | 0.49 (0.44–0.55)^***^ |
| **Sex** | Male | *Ref* | *Ref* |
|  | Female | 1.35 (1.25–1.47)^***^ | 1.36 (1.25–1.47)^***^ |
| **Age^a^** |  | 1.07 (1.06–1.08)^***^ | 1.07 (1.06–1.08)^***^ |
| **Birth cohort** | Post-war (1954-1961) | *Ref* | *Ref* |
|  | Japanese annexation (1932-1944) | 1.07 (0.97–1.18) | 1.07 (0.97–1.18) |
|  | Korean liberation (1945-1949) | 1.19 (1.08–1.31)^***^ | 1.19 (1.08–1.31)^***^ |
|  | Korean War (1950-1953) | 1.11 (1.00–1.23) | 1.11 (1.00–1.23) |
| **Age:Birth cohort interaction** | Age:Post-war (1954-1961) | *Ref* | *Ref* |
|  | Age:Japanese annexation (1932-1944) | 0.99 (0.98–1.00)^*^ | 0.99 (0.98–1.00)^*^ |
|  | Age:Korean liberation (1945-1949) | 0.99 (0.98–0.99)^***^ | 0.99 (0.98–0.99)^***^ |
|  | Age:Korean War (1950-1953) | 0.99 (0.98–1.00)^*^ | 0.99 (0.98–1.00)^*^ |
| **Parental death during childhood** | No |  | *Ref* |
|  | Yes |  | 1.10 (1.01–1.20)^*^ |
| **Parental education** | No formal education |  | *Ref* |
|  | Elementary school |  | 1.02 (0.95–1.10) |
|  | Middle school or more |  | 0.98 (0.89–1.09) |
| **Own education** | Middle school or less | *Ref* | *Ref* |
|  | High school | 0.80 (0.74–0.86)^***^ | 0.80 (0.74–0.87)^***^ |
|  | College/university | 0.69 (0.62–0.77)^***^ | 0.70 (0.62–0.78)^***^ |
| **Place of residence** | Urban | *Ref* | *Ref* |
|  | Rural | 0.90 (0.85–0.96)^***^ | 0.91 (0.86–0.96)^***^ |
| **Smoking status** | Never smoker | *Ref* | *Ref* |
|  | Ever smoker | 1.14 (1.05–1.24)^**^ | 1.14  (1.05–1.24)^**^ |
| **Random intercept variance** |  | 1.29 | 1.28 |
| **Intraclass Correlation Coefficient (ICC)** |  | 0.70 | 0.70 |

*Note:* Results were computed using Poisson mixed-effects models with repeated measures at level 1 and individuals at level 2. Due to rounding, some confidence intervals appear to include 1.00 but remain statistically significant.

^a^ Age is grand-mean centered at 62.9 years.

^*^p<0.05, ^**^p<0.01, ^***^p<0.001
